# Subcellular patterns of SPE-6 localization reveal unexpected complexities in *Caenorhabditis elegans* sperm activation and sperm function

**DOI:** 10.1093/g3journal/jkab288

**Published:** 2021-10-16

**Authors:** Jackson J Peterson, Claire E Tocheny, Gaurav Prajapati, Craig W LaMunyon, Diane C Shakes

**Affiliations:** 1 Department of Biology, William & Mary, Williamsburg, VA 23187, USA; 2 Department of Biological Science, California State Polytechnic University, Pomona, CA 91768, USA

**Keywords:** *spe-6*, casein kinase I, sperm activation, spermiogenesis, sperm–uterine interactions, *Caenorhabditis elegans*, perinuclear

## Abstract

To acquire and maintain directed cell motility, *Caenorhabditis elegans* sperm must undergo extensive, regulated cellular remodeling, in the absence of new transcription or translation. To regulate sperm function, nematode sperm employ large numbers of protein kinases and phosphatases, including SPE-6, a member of *C. elegans*’ highly expanded casein kinase 1 superfamily. SPE-6 functions during multiple steps of spermatogenesis, including functioning as a “brake” to prevent premature sperm activation in the absence of normal extracellular signals. Here, we describe the subcellular localization patterns of SPE-6 during wild-type *C. elegans* sperm development and in various sperm activation mutants. While other members of the sperm activation pathway associate with the plasma membrane or localize to the sperm’s membranous organelles, SPE-6 surrounds the chromatin mass of unactivated sperm. During sperm activation by either of two semiautonomous signaling pathways, SPE-6 redistributes to the front, central region of the sperm’s pseudopod. When disrupted by reduction-of-function alleles, SPE-6 protein is either diminished in a temperature-sensitive manner (*hc187*) or is mislocalized in a stage-specific manner (*hc163*). During the multistep process of sperm activation, SPE-6 is released from its perinuclear location after the spike stage in a process that does not require the fusion of membranous organelles with the plasma membrane. After activation, spermatozoa exhibit variable proportions of perinuclear and pseudopod-localized SPE-6, depending on their location within the female reproductive tract. These findings provide new insights regarding SPE-6’s role in sperm activation and suggest that extracellular signals during sperm migration may further modulate SPE-6 localization and function.

## Introduction

For sperm, cell motility is a tightly regulated and multifaceted process. Within males, sperm are maintained in a quiescent state, but after transfer to the female reproductive tract, they acquire motility and migrate directionally to the oocytes (Smith and Stanfield 2011). Precocious sperm activation squanders metabolic resources and inhibits sperm transfer, while delays in sperm activation place individual sperm at a competitive disadvantage for fertilization events (Hirohashi *et al.* 2016). To optimize their own fertility, females of many species have evolved the capacity to store sperm either in pouch-like receptacles called spermatheca (Marcello *et al.* 2013; Pascini and Martins 2017) or within specialized regions of the oviduct where sperm viability is prolonged by repressing sperm motility and capacitation (Suarez 2008; Breton *et al.* 2016). However, the molecular mechanisms that regulate sperm activation remain incompletely understood (Ellis and Stanfield 2014; Ritagliati *et al.* 2018).

**Figure 1:**
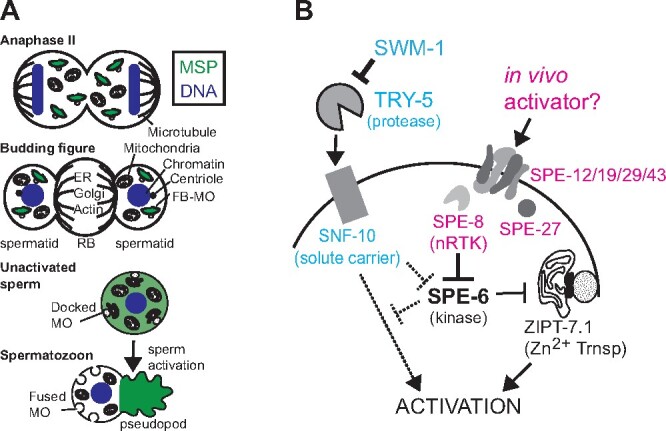
Overview of postmeiotic sperm development and sperm activation. (A) Schematic of key developmental transitions including (1) the differential partitioning of organelles between the spermatids and the central RB, 2) the release of the MSP (green) from the FB as the MOs dock with the plasma membrane of newly separated spermatids, and (3) the two key events of sperm activation: formation of an MSP-powered pseudopod and the fusion of the MOs with the plasma membrane in a manner that generates a stable fusion pore. (B) Model of the male (blue) and hermaphrodite (pink) activation pathways based on data from the current literature with experimentally supported interactions indicated by solid black lines. It is currently unclear whether or what point the two pathways merge but hypothetical and yet to be elucidated pathways are indicated with dotted lines. The male pathway includes a protease that is secreted during ejaculation from the vas deferens but which is inhibited by the protease inhibitor SWM-1. The extracellular signal for the hermaphrodite pathway is unknown but the pathway includes the transmembrane proteins (SPE-12/19/29/43), the nontransmembrane protein SPE-27, and the nonreceptor tyrosine kinase SPE-8. Other key factors including ZIPT-7.1 localize to the membranous organelle.

In the androdiecious (male/hermaphrodite) nematode *Caenorhabditis elegans*, both sperm production and sperm activation are streamlined events. Developing spermatocytes synthesize and prepackage complexes and organelles needed by the mature sperm (Chu and Shakes 2013). Transcription ceases prior to the meiotic divisions (Shakes *et al.* 2009). Translation ceases after anaphase II, as differentiated but nonmotile sperm are generated through an asymmetric partitioning process. During this process, haploid sperm retain their chromatin, mitochondria, and sperm-specific fibrous body—membranous organelle (FB-MO) complexes while discarding both their biosynthetic machinery (ER, Golgi, & ribosomes) and standard cytoskeletal proteins (actin and tubulin) into a central residual body (RB) (Figure 1A; Ward *et al.* 1981; Ward 1986; Winter *et al.* 2017). Once spermatids detach from the RB, their MOs dock with the plasma membrane and their FBs disassemble to release the major sperm protein (MSP) into the cytosol (Ward *et al.* 1981; Chu and Shakes 2013). Sperm activation is a distinct event during which spherical, non-motile spermatids are converted into crawling spermatozoa. Sperm activation is defined by two irreversible cellular events: the Golgi-derived, acrosome-analogous MOs fuse with the cell membrane and the cell polarizes to extend a pseudopod whose motility is driven by cycles of MSP assembly/disassembly (Ellis and Stanfield 2014).

Members of the *Caenorhabditis* genus utilize two semiautonomous but likely converging sperm activation pathways; males redundantly use both pathways to activate their sperm (Shakes and Ward 1989; Smith and Stanfield 2011; Ellis and Stanfield 2014), whereas hermaphroditic species have independently evolved to co-opt one of the two pathways (Wei *et al.* 2014; Figure 1B). In *C. elegans*, self-fertilizing hermaphrodites activate their sperm via the nonreceptor tyrosine kinase (SPE-8), and several additional sperm-specific proteins, collectively called the *spe-8* group (L’Hernault 2009; Muhlrad *et al.* 2014; Krauchunas *et al.* 2018; Geldziler *et al*. 2005). Of these *spe-8* group members, the two transmembrane proteins, SPE-12 and SPE-19, are required to localize SPE-8 to the plasma membrane in spermatids (Muhlrad *et al.* 2014). Parallel to the *spe-8* pathway, a second, redundant activation pathway was revealed by the fertility of *spe-8* group males and the observation that *spe-8* hermaphrodite sperm are cross-activated following copulation (Shakes and Ward 1989). Subsequent genetic studies identified the serine protease TRY-5 as a factor in male seminal fluid that acts directly or indirectly through the SLC6 type transporter SNF-10 to induce activation in a *spe-8* independent manner (Smith and Stanfield 2011; Fenker *et al.* 2014). Regulated sperm activation is critical to fertility; within male gonads, the protease inhibitor SWM-1 actively prevents TRY-5 from precociously activating stored sperm within the seminal vesicle (Stanfield and Villeneuve 2006). Exactly how the intracellular components of either pathway subsequently transmit the signal remains poorly understood, but the involvement of ion fluxes and pH changes are implicated by the success of *in vitro* activators like the weak base triethanolamine and the ionophore monensin (Nelson and Ward 1980; Shakes and Ward 1989). Most recently, divalent zinc has been proposed to function downstream of both the *spe-8* and* try-5/snf-10* pathways; new studies suggest that the MO localized zinc transporter ZIPT-7.1 functions late in the pathway to promote sperm activation through the regulated release of intracellular zinc (Liu *et al.* 2013; Zhao *et al.* 2018; Figure 1).

Genetic studies suggest that the ser/thr kinase SPE-6 functions near the end of both pathways. *spe-6* is one member of a greatly expanded, *C. elegans’* casein kinase I superfamily (CK1); *C. elegans* has 78 genes versus 12 in humans (Plowman *et al.* 1999; Manning 2005; Fulcher and Sapkota 2020). In humans, the CK1 group consists of three main subfamilies: CK1 (7 genes), VRK (Vaccinia-Related Kinase; 3 genes), and TTBK (Tau Tubulin Kinase; 2 genes). Of these, SPE-6 is more closely related to TTBK; however, SPE-6 actually belongs to a distinct “worm 6” subgroup of 28 members (Manning 2005; Andrade *et al.* 2011). Many of these are expressed in a spermatogenesis-enriched manner and may be functionally redundant (Muhlrad and Ward 2002; Reinke *et al.* 2000); however, *spe-6* itself is essential for multiple steps in spermatogenesis. In *spe-6* null mutants, spermatocytes not only fail to assemble FBs but are also unable to complete their meiotic divisions (Varkey *et al.* 1993; Price *et al.* 2021). A second, later function for SPE-6 was defined by the many non-null, loss-of-function alleles that were isolated as suppressors of *spe-8* class mutants (Muhlrad and Ward 2002). These *spe-6* alleles have two phenotypes. In double-mutant combinations, they restore self-fertility to *spe-8* class hermaphrodites (Muhlrad and Ward 2002). In homozygous mutant males, sperm activate precociously, independent of normal extracellular signals (Muhlrad and Ward 2002). Such males are infertile, presumably since precocious sperm activation within the male seminal vesicle disrupts sperm transfer during mating. These phenotypes suggest that SPE-6 functions within spermatids to repress precocious sperm activation.

Here, we use a newly generated anti-SPE-6 antibody to analyze the patterns of *spe-6* protein localization during spermatogenesis and the subsequent events of sperm activation. We find that the protein is first detectable in cytoplasmic speckles within developing spermatocytes but, following the meiotic divisions, SPE-6 specifically surrounds the chromatin mass of haploid spermatids. In the canonical and most robust suppressor allele *spe-6*(*hc163*) SPE-6 failed to either properly partition to the spermatids following anaphase II or subsequently localize around the sperm’s chromatin mass in a perinuclear fashion. In contrast, *spe-6*(*hc187*) mutants were found to be temperature-sensitive for *spe-6* protein levels. During both *in vivo and in vitro* sperm activation, wild-type SPE-6 begins to redistribute from its perinuclear localization to the pseudopod. Within spermatozoa, the relative distribution of perinuclear and pseudopod localized SPE-6 is highly variable and may depend on the physical location of spermatozoa within the reproductive tract of the hermaphrodite. Not only do these findings provide new insights into *C. elegans* sperm activation but they confirm *C. elegans* as a powerful model for studying sperm function and development.

## Materials and methods

### *Caenorhabditis elegans* genetics and culturing

*Caenorhabditis* *elegans* genetics and husbandry were accomplished using standard methods (Brenner 1974). Worms were cultured on MYOB plates seeded with OP50 strain of *Escherichia* *coli* and maintained at 22°C unless otherwise noted. For the ease of obtaining males, many experiments were done in animals containing homozygous or heterozygous *him-5* or *him-8* mutations; these *him* (high incidence of males) mutations increase male production by increasing meiotic nondisjunction of the X chromosome; yet the animals produce normal sperm. In characterizing the antibody and mating studies, XX *fog-2* (feminization of germline) hermaphrodites were used as “females” as these mutants produce normal oocytes but fail to produce self-sperm. Some *C. elegans* strains were acquired from the Caenorhabditis Genetics Center. Strains used were all derived from N2 Bristol and were as follows: CB1489 *him-8(e1489)* IV. BA984 *spe-6(hc163) dpy-18(e364)* III. ZQ130 *spe-6(hc187)* III; *him-5*(*e1490*) V*.* BA606 *spe-6(hc49) unc-25(e156)* III; *eDp6* (III; f). BA784 *spe-8(hc50) I*. BA900 *spe-27(it110)* IV. AV322 *swm-1(me87) him-5(e1490)* V. BA1093 *snf-10(hc193).* DS6 *emb-27(g48ts)* II; *him-5(e1490)* V. BA524 *fer-1(hc1)* I; *him-5(e1490)* V. CB4108 *fog-2(q71)* V. ZQ170 *spe-6*(*hc187*) III; *spe-29*(*it127*) *dpy-20*(*e1282*) IV.

### Analysis of male and hermaphrodite fertility

To analyze the fertility of *spe-6(hc187)* males, males were either reared continuously at 16°C or upshifted to 25°C as L2/L3 larvae. Cross plates were set up with six young adult males and three celibate, young adult *fog-*2 “females” at 16°C or 25°C as appropriate, and then the worms were allowed to mate overnight. Inseminated females were then transferred without males to individual 25°C plates and thereafter transferred to fresh 25°C plates daily until they were no longer producing embryos. Daily progeny counts were made as soon as those embryos had reached the L4 or young adult stage. *fog-*2 males were used as controls.

To analyze the self-fertility of *spe-6(hc187)* hermaphrodites, worms were maintained at 16°C or upshifted to 25°C as L2/L3 larvae. Individual hermaphrodites were then picked as L4 larvae to individual, temperature-acclimated plates. A subset of the worms raised as 16°C through larval development were upshifted to 25°C as young adults just after they laid their first few embryos. The individual adults were then transferred to fresh plates daily to obtain total brood counts as detailed above.

### Immunocytology

**Figure 2:**
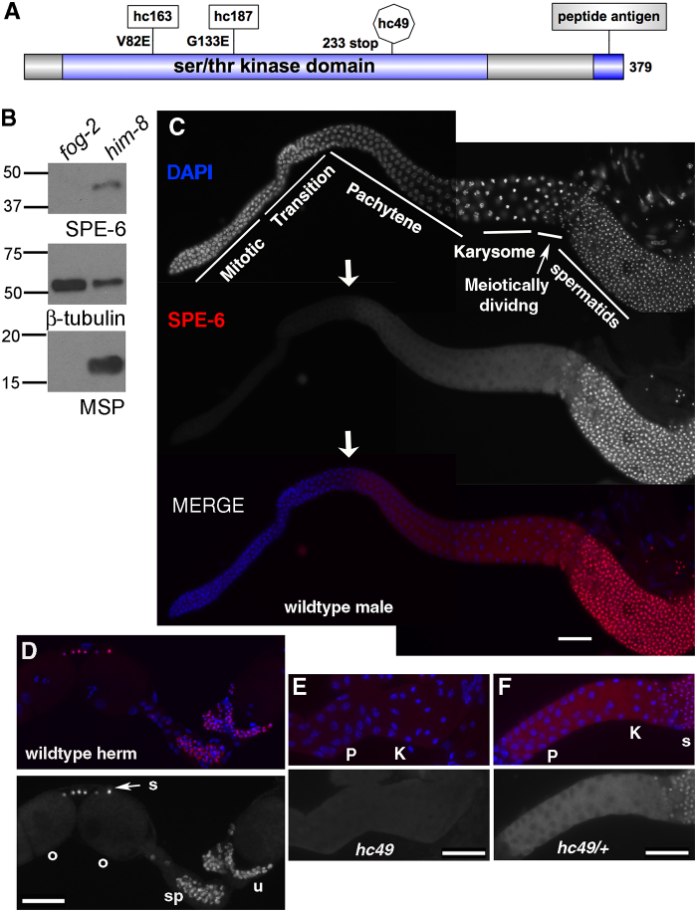
SPE-6 polyclonal antibody specifically labels developing spermatocytes and haploid sperm. (A) Cartoon schematic including the site of the peptide used for antibody generation and the molecular lesions of the mutant *spe-6* alleles used in this paper. (B) Western blot of SPE-6 in *him-8* (wild-type proxy) males and *fog-2* “females.” Loading controls of β-tubulin and the MSP. (C–F) Isolated gonads stained with DAPI(blue) and SPE-6 (red). Animals were dissected and processed on same slide and imaged with identical exposures. (C) Full gonad of wild-type adult male with subregions labeled and arrow indicating the start of SPE-6 expression. (D) Region of wild-type adult hermaphrodite gonad with oocytes (o) on the left, unactivated spermatids in the oviduct (s), and spermatozoa (sp) in the spermatheca and adjacent region of the uterus (u). The gonad is outlined for clarity. (E and F) Regions of isolated male gonads with pachytene (P), karyosome (K), and spermatid (s) regions indicated. (E) *spe-6(hc49*) homozygote of null allele with a premature stop codon that deletes the antigenic site. (F) *spe-6*(*hc49*)/+ heterozygous sibling. In all images, distal is to the left. Scale bars = 25 μm.

Whole gonads were obtained with individual dissections in egg buffer (Edgar 1995) and in some cases, sperm spreads were obtained by applying additional pressure to greased coverslips (Golden *et al.* 2000). For methanol fixation, samples were flash-frozen in liquid nitrogen and fixed in cold −20°C methanol for at least 1 day. Fixed specimens were incubated at room temperature with primary and secondary antibodies for 1.5–2 h. The following primary antibodies were used: mouse monoclonal anti-MSP (1:1500; 4D5, gift from D. Greenstein), mouse monoclonal 1CB4 (1:150; Gift from Steve L’Hernault), and anti-SPE-6 (1:250; Yenzyme). Rabbit anti-SPE-6 polyclonal antibody was generated by Yenzyme against the following C-terminal peptide epitope (360–379): KSLSAEKSCTKNVETARTEK (Figure 2A; Yenzyme). SPE-6 antisera was affinity purified using the peptide antigen (Yenzyme). The following affinity-purified commercial secondary antibodies were used: Dylite labeled anti-mouse (1:100; Jackson Immunoresearch Laboratories), TRITC labeled anti-rabbit (1:800; Jackson Immunoresearch Laboratories). Images were taken in the linear range, and in some cases, Adobe Photoshop was used to linearly adjust images for clarity. Any nonlinear adjustments for purposes of increased contrast are specifically noted in the figure legend.

### Sperm activation counts

Synchronized worms were maintained at the indicated temperature starting at an embryonic or L1 stage. Males were picked away from hermaphrodites at the L4 stage to ensure male celibacy. Two days after isolation, or 1 day after isolation at 25°C to reflect temperature-dependent metabolic differences, males were dissected as above and prepared for indirect immunofluorescence against MSP. Slides were blind scored; each slide was scored for the number of worms exhibiting precocious activation based on the presence or absence of MSP positive pseudopods. Only animals with a large (>50) field of spermatids/spermatozoa were scored. The benchmarks of 0–1%, 1–5%, and >5% precociously activated spermatozoa were used in scoring to make our system sensitive to any level of precocious activation.

### Western blots

For western blot experiments requiring isolated males, 80 worms (except in Figure 1 which contained 300 males of each genotype and 300 feminized hermaphrodites) were collected in 20 µL of M9 buffer containing protease inhibitor (Roche 11 836 170 001) in the cap of an Eppendorf tube. Samples were flash-frozen in liquid nitrogen and stored at −80°C.

Frozen samples were homogenized with 20-µL sample buffer (Laemmli Sample Buffer, Bio-Rad) containing 5% beta-mercaptoethanol (MP Biomedicals) heated to 95°C, boiled for 5 min, and centrifuged for 3 min at 15,000 relative centrifugal force (rcf). SDS-PAGE was performed on lysates from 80 to 200 worms at 150 V (Mini-Protean^®^ TGX Gels, Bio-Rad) and transferred to a polyvinylidene difluoride membrane using a semi-dry protocol (GE Healthcare). Membranes were blocked overnight with Tris-buffered saline with Tween 20 (TBST; 0.2M Tris HCl pH 8.0, 1.5M NaCl, 0.1% Tween 20) containing 4% nonfat dry milk (Carnation).

After blocking, membranes were incubated with the appropriate antibody diluted in blocking buffer (4% milk (w/v) in 1X TBST) for 2 h at room temperature. Following 2-h antibody incubations, membranes were washed for 15 min six times in 1x TBST. The following primary antibodies and concentrations were used for western blotting: HRP conjugated (Abcam) anti-SPE-6 YZ2698 rabbit polyclonal (Yenzym) at a 1:10,000 dilution and anti-MSP mouse monoclonal 4D5 at a 1:50,000 dilution. MSP blots were probed with an HRP conjugated polyclonal goat-anti-mouse secondary at a 1:10,000 dilution (Jackson Laboratories). Antibodies were detected with enhanced chemiluminescence (Immobilon™ Western Chemiluminescent HRP Substrate, Millipore), exposed to X-ray film, and developed. For additional reagent details see Supplementary Reagent Table.

### *In vitro* sperm activation

Celibate aged male worms were dissected on Colorfrost Plus slides (Shandon) in egg buffer (Edgar, 1995) containing 200 µg/mL protease from *Streptomyces* *grieus* (pronase; Sigma) with care made to attempt to get large visible “streaks” of spermatids to stick to the positively charged slides. Pronase efficacy was verified by differential interference contrast (DIC) microscopy on wild-type sperm during each independent experiment, and attempts were sometimes made to take DIC pictures at specific coordinates to match to later immunofluorescence images. Times given are the time since the last cut was made during dissection, so the times given are the minimum time that the sperm were sitting in dissection solution with a variation of up to approximately 3 min.

To assess the localization of SPE-6 in male *in vivo* activated sperm, wild-type young adult males were plated with L4 *fog-2(q71)* feminized hermaphrodites for 24 h. These mated feminized hermaphrodites were dissected (with attempts made to lay out spermathecae) and flash-frozen for immunofluorescence as above.

## Results

### Anti-SPE-6 antibody reveals stage-specific patterns of SPE-6 localization

To better understand how SPE-6 regulates sperm function, we constructed a polyclonal antibody against a C-terminal peptide sequence (Figure 2A; see *Materials and* *Methods*). Consistent with transcriptomic studies, our antibody bound a protein at the expected molecular weight of 42 KDa in *him-8* (wild type) males but not in *fog-2* “females” (Figure 2B; Reinke *et al.* 2000; Ortiz *et al.* 2014). In isolated male gonads, SPE-6 antibody labeling was first detectable in the cytoplasm of spermatocytes during the early pachytene stage of meiotic prophase (2C, arrow), and then prominently associated with the hyper-condensed chromatin masses of haploid spermatids. In adult hermaphrodites, the antibody labeled both unactivated spermatids and crawling spermatozoa, but not oocytes (2D). In an additional control, homozygous males of a *spe-6* null allele (*hc49*), which encodes a version of SPE-6 that truncates translation prior to the antigenic site and functions as a genetic null, exhibited only background levels of signal (2E) whereas heterozygous siblings (2F) exhibited wild-type patterns.

**Figure 3:**
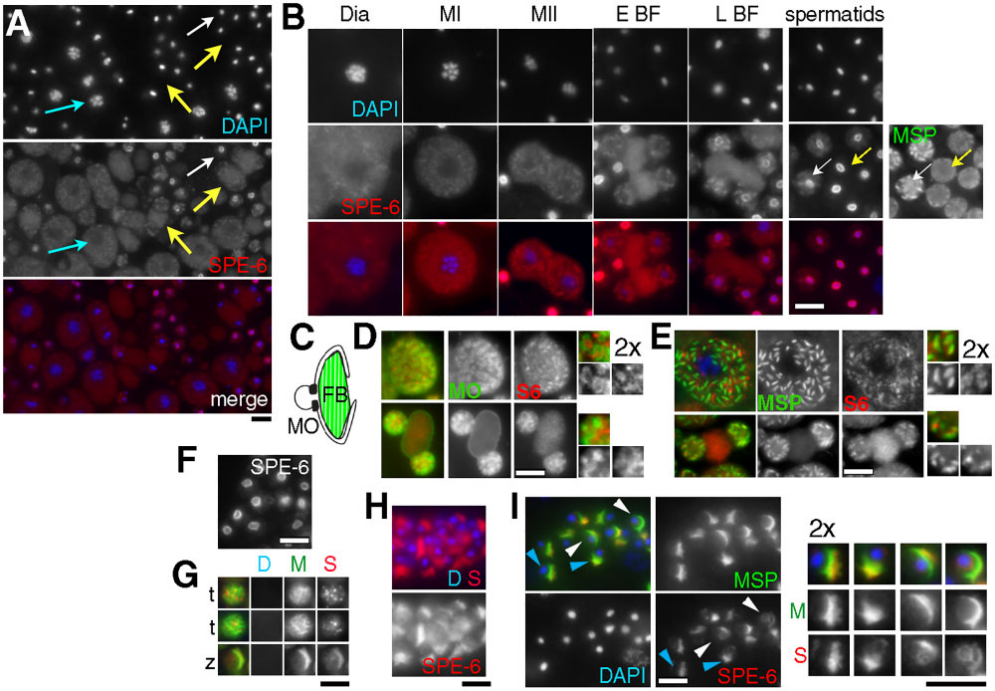
Stage-specific, subcellular patterns of SPE-6 localization. (A) Monolayer of spermatocytes (blue arrow), early and late budding figures (yellow arrows), and spermatids (white arrow) from wild-type male labeled with DAPI and anti-SPE-6 antibody. (B) Stage-specific patterns. Diakinesis (Dia), metaphase I (MI), metaphase II (MII), early budding figure (E BF) late budding figure (L BF). Immature (white arrow) and mature (yellow arrow) spermatids colabeled with anti-MSP. (C) Schematic of an FB-MO complex. (D and E) Spermatocytes (top) and BF (bottom) colabeled with anti-SPE-6 and either the MO marker 1CB4 (D) or the FB marker MSP (E). Side images are 2X enlarged and contrast enhanced. (F) SPE-6 labeled spermatids from immuno-preparation without DAPI. (G) Anucleate spermatids (t) and spermatozoon (z) from the temperature-sensitive anaphase-promoting complex mutant *emb-27*(*g48*) colabeled with DAPI (D), MSP (M), and SPE-6 (S). (H and I) Spermatozoa within the spermathecae of hermaphrodites labeled with SPE-6 alone (H) or colabeled with MSP (I). Male sperm (H) are larger than hermaphrodite sperm (I). 2X enlarged images of single spermatozoon are shown for clarity. SPE-6 often labels a smaller region of the pseudopod than MSP (blue arrowheads) or is present in both the pseudopod and the perinuclear halo (white arrowheads). SPE-6 (red), DNA (blue), and MSP (green). Scale bars = 5 μm.

To further examine the subcellular patterns of SPE-6 localization and how these patterns might change during spermatogenesis and subsequent sperm development, we examined SPE-6 localization patterns in wild-type male gonads that had been flattened to generate a cellular monolayer. Within these gonads, the subcellular localization pattern of SPE-6 changed at several key transitions during spermatogenesis, shifting from a particulate plus diffuse cytoplasmic pattern in developing spermatocytes to a perinuclear pattern around the chromatin of unactivated spermatids (Figure 3, A and B). The nature of the structured particulate pool remains unclear as SPE-6 failed to colocalize with either MO or FB markers (Figure 3, C–E). The initial spermatocyte pattern persisted until anaphase II. During the subsequent partitioning phase, the particulate SPE-6 structures partitioned to the sperm while the diffuse SPE-6 pool concentrated within the RB (Figure 3B). As spermatids separated from the RB, the particulate pool of SPE-6 redistributed to surround the haploid chromatin mass. In most individualized sperm, SPE-6 localized exclusively around the chromatin. To test whether rare sperm with both cytoplasmic and chromatin-associated SPE-6 corresponded to newly formed spermatids, spermatids were colabeled with antibodies against MSP (Figure 3B, side image). Indeed, these sperm (white arrow) had yet to fully disassemble their MSP-containing FBs. To clarify whether SPE-6 was concentrating around rather than binding directly to the chromatin, we examined non-DAPI stained preparations and found that the central region of chromatin mass remained unlabeled, suggesting that the observed SPE-6 pattern was not an artifact of the antibody signal being quenched by DAPI staining (Figure 3F). Consistent with this idea, in mutant spermatids that lack DNA because they aberrantly discard their chromatin into the RB (Golden *et al.* 2000; Sadler and Shakes 2000), SPE-6 localized to multiple, discrete cytoplasmic particles (Figure 3Gt). For simplicity, we will henceforth refer to the wild-type SPE-6 pattern in unactivated spermatids as perinuclear, although the haploid chromatin mass of *C. elegans* spermatids technically lacks a nuclear envelope and is instead encased in an electron-dense material that stains positively for RNA (Wolf *et al.* 1978).

To determine the SPE-6 pattern in spermatozoa, we examined labeled sperm within the spermathecae of hermaphrodites. Whether spermatozoa were labeled with anti-SPE-6 alone (Figure 3H) or colabeled with MSP (Figure 3I), SPE-6 localized primarily to the pseudopod (blue arrowheads). Importantly, many spermatozoa retained detectable levels of perinuclear SPE-6 labeling (white arrowheads), even when MSP localized exclusively to the pseudopod. Within the pseudopod, the SPE-6 and MSP patterns either fully overlapped or SPE-6 was restricted to a smaller region of the pseudopod, either at the leading edge or in the more central region of the pseudopod (Figure 3I, enlarged side images ). Despite this variability, there was a clear difference between the exclusively perinuclear pattern of SPE-6 in unactivated spermatids and the mostly pseudopod pattern of SPE-6 in spermatozoa. Furthermore, the absence of a nucleus did not alter this shift of SPE-6 to the pseudopod (Figure 3Gz).

### *spe-6(hc187)* but not *spe-6(hc163)* is temperature-sensitive for precocious sperm activation and *spe-6* protein levels

Given SPE-6’s purported role as an inhibitor of precocious sperm activation, the finding that SPE-6 localized to the perinuclear region of unactivated spermatids was a surprise. *A priori*, we had predicted that SPE-6 would localize to either the plasma membrane or plasma membrane docked MOs where other known components of the sperm activation pathway reside (reviewed in Arduengo *et al.* 1998; Gosney *et al.* 2008; Ellis and Stanfield 2014; Zhao *et al.* 2018). Although genetic evidence supported categorizing *spe-6* suppressor class alleles as recessive, loss-of-function alleles (Muhlrad and Ward 2002), the perinuclear localization pattern of SPE-6 made us reconsider whether these special non-null *spe-6* alleles might be neomorphic rather than reduction of function alleles.

**Figure 4:**
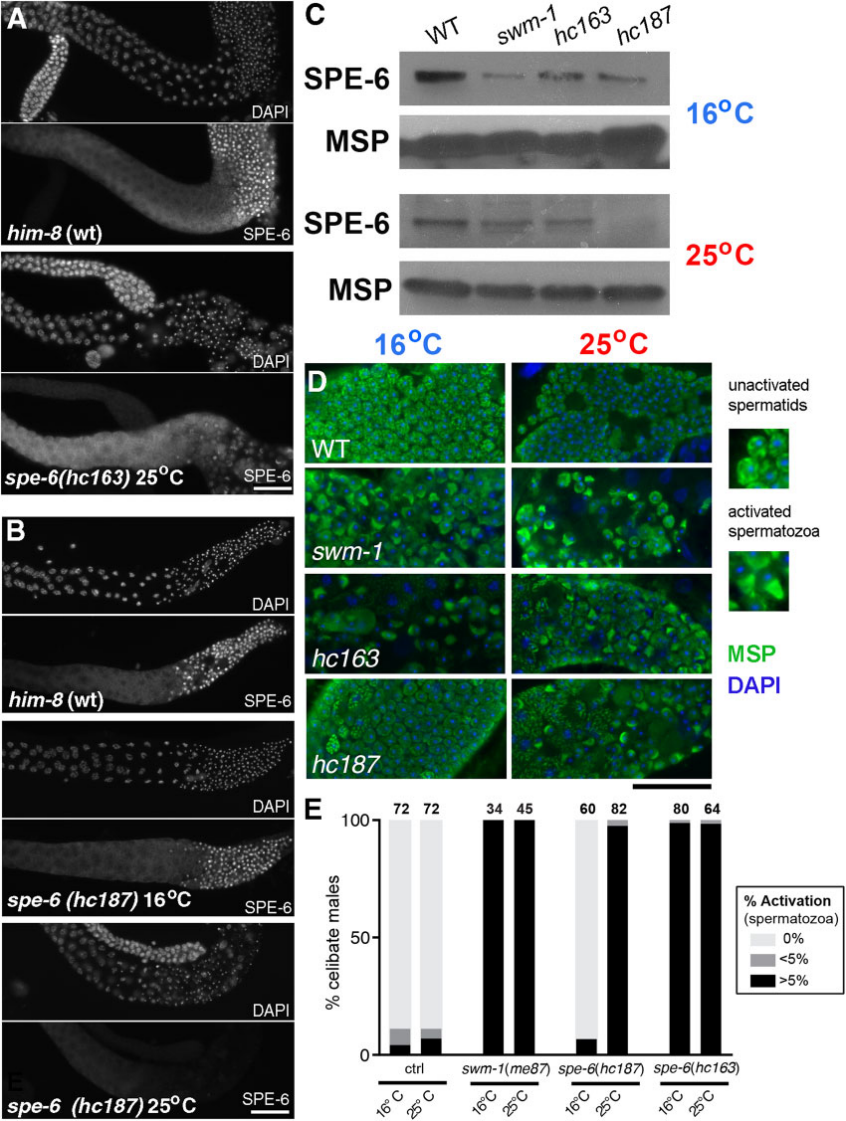
*spe-6(hc187)*, but not *spe-6(hc163)* is temperature-sensitive for precocious sperm activation and *spe-6* protein levels. (A and B) Wild type and *spe-6* male gonads of the indicated allele stained with DAPI (Top) and SPE-6 (Bottom). Males were raised from embryos or larval stage 1 (L1) at the indicated temperature, picked separate from hermaphrodites as L4s to ensure celibacy, and processed for immunofluorescence at the same time. (C) Representative western blots of wild type and mutant whole worm lysates from 80 same-age adults raised at the indicated temperature. Membranes probed with anti-SPE-6 antibody yielded a band at approximately MW of 42 kDa. Anti-MSP (MW = 15 kDa) was used as a sperm protein loading control. (D) Epifluorescence micrographs of representative sperm spreads from celibate males stained with DAPI(blue) and MSP(green). Enlarged images below highlight the distinct patterns of MSP in spermatids and spermatozoa. (E) Gonads of celibate males were scored for the presence of activated spermatozoa. As per published activation studies, each gonad was categorized as containing less than 1% (unactivated), between 1% and 5%, and greater than 5% activated spermatozoa. *N* values are displayed above each bar. Scale bars = 25 µm.

To distinguish between these two possibilities, we sought to determine if one or more of these non-null *spe-6* alleles had reduced levels of *spe-6* protein. For these studies, we chose to examine both the canonical allele *hc163* with its valine to glutamic acid substitution at a conserved residue in the N-terminal ATPase domain and a second allele *hc187* whose specific alteration, a G to E substitution in a conserved tau tubulin kinase residue, had been identified in two independently isolated alleles (Figure 2A; Muhlrad and Ward 2002). Isolated gonads from *spe-6*(*hc163*) males routinely exhibited high levels of SPE-6 in developing spermatocytes and low or variable levels of SPE-6 in haploid sperm (Figure 4A). In contrast, *spe-6(hc187)* proved to be temperature-sensitive. Gonads from *hc187* males raised and maintained at 16°C exhibited wild-type levels and patterns of SPE-6 whereas SPE-6 levels in *hc18*7 males raised at 25°C were drastically reduced (Figure 4B). In parallel western blot studies (Figure 4C), celibate *spe-6*(*hc163)* males raised at either 16°C or 25°C produced SPE-6 of a molecular weight and at levels comparable to both celibate wild-type males (which accumulate unactivated spermatids) and *swm-1(me87)* males (which accumulate spermatozoa due to the lack of an extracellular protease inhibitor; Stanfield and Villeneuve 2006). In contrast, *spe-6*(*hc187)* produced drastically reduced levels of SPE-6, specifically when raised and maintained at 25°C. These results reveal *spe-6(hc187)* as a temperature-sensitive, reduction of function mutant that produces dramatically less SPE-6 at the restrictive temperature. In addition, they confirm the specificity of our antibody and suggest that minimal amounts of SPE-6 are sufficient to carry out its essential early functions within developing spermatocytes.

Because a complete loss of SPE-6 results in meiotically arrested spermatocytes, it had been difficult to assert that the absence of SPE-6 in unactivated spermatids was sufficient to induce precocious sperm activation. However, identification of *hc187* as a temperature-sensitive, loss-of-function allele provided us with a critical tool for directly addressing this question. Gonads from celibate males raised at either the permissive or restrictive temperature were examined by immunofluorescence and scored for precocious activation. We observed striking differences between the two conditions. When raised at 25°C, all *hc187* males analyzed exhibited precocious activation. However, in those raised at 16°C, <5% exhibited precocious activation (Figure 4, D and E). The elevated temperature did not modulate precocious activation in *him-8* (control), *swm-1*, or *spe-6(hc163)* males, confirming that the temperature-sensitive phenotype is specific to *spe-6*(*hc187).* Therefore, these studies lend support to the contention that low SPE-6 levels result in precocious activation and to the hypothesis that, in wild-type sperm, SPE-6 functions as a “brake” to sperm activation.

**Figure 5:**
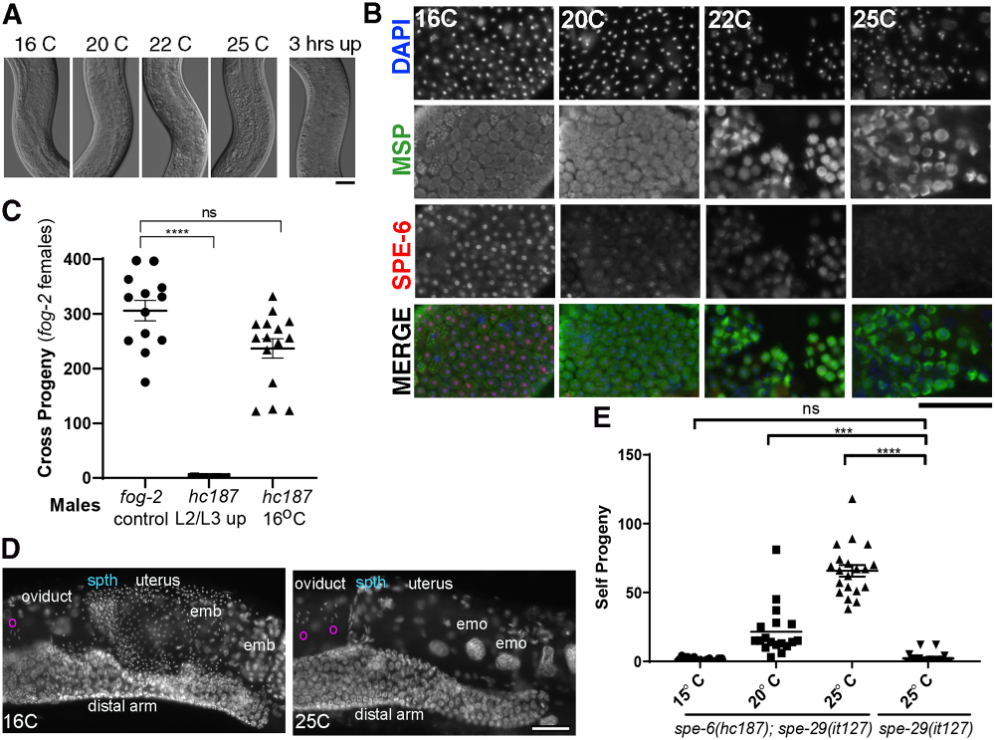
Characterization of *spe-6*(*hc187*). Precocious sperm activation in *spe-6(hc187)* at intermediate temperatures. (A) DIC micrographs of the seminal vesicle of celibate, whole mounted *spe-6(hc187)* males raised continuously at the indicated temperature or raised at 16°C and upshifted to 25°C for 3 h. Seminal vesicles containing unactivated spermatids have a smooth appearance relative to those with spermatozoa. (B) Sperm spreads from sibling worms labeled with both DAPI(blue) and antibodies against the MSP (green) and SPE-6(red). Exposures were not standardized. (C) Male fertility assessed by brood sizes of *fog-2* “females” crossed for 12 h with *fog-2* control males or *hc187* males that were upshifted from 16°C to 25°C either before spermatogenesis (L2/L3 larval upshift) or after spermatogenesis (adult up). (D) Analysis of male sperm transfer. Images of whole-body DAPI-stained *fog-2* females that were mated overnight to *spe-6*(*hc187*) males raised at 16°C or 25°C as noted in C. Numerous tiny, DAPI-bright dots that fill the spermatheca and uterus in the 16°C cross are the condensed sperm nuclei. Labeled regions include the distal arm of the gonad, the oviduct, oocytes (o), spermatheca (spth), uterus, embryos (emb), and unfertilized endomitotic (emo) oocytes. (E) Temperature-dependent suppression of *spe-29(it127)* hermaphrodite self-fertility in *spe-6(hc187)* double mutants. For ease of the analysis, *spe-29* was linked to the genetic marker *dpy-20*(*e1282*). Scale bars = 25 µm. (C, E) Statistical analysis based on Kruskal–Wallis test with Dunn’s post-test and using Graphpad Prism V8.1.2. Bars depict mean and SEM. Statistical comparisons proved to be either nonsignificant (ns) or significant at the level of *P* < 0.001 (***) or *P* < 0.0001(****).

In additional experiments, we further characterized the temperature-sensitive phenotype of *spe-6*(*hc187*) mutants and assessed how depleting SPE-6 affected sperm function. (1) Analysis of *spe-6*(*hc187*) males raised at various temperatures revealed that the critical temperature for precocious activation is between 20°C and 22°C (Figure 5, A and B). The observed phenotypic differences were strikingly robust and provided no evidence of an intermediate phenotype. (2) Shifting *hc187* adult males that had been raised at 16°C to 25°C for 3 h failed to induce widespread sperm activation (Figure 5A); a result that suggests that the mutant protein is temperature-sensitive for synthesis rather than function and that already synthesized *spe-6*(*hc187*) protein is not rapidly inactivated by increased temperatures. (3) In studies to assess male fertility, *fog-2*(*q71*) “females” (Schedl and Kimble 1988) were crossed with males and then the inseminated females were isolated and maintained at 25°C. *spe-6(hc187); him-8* males reared at 16°C sired statistically similar numbers of progeny as *fog-2* control males, whereas *spe-*6(*hc187); him-8* males that had been upshifted to 25°C as L2/L3 larvae sired no or very few progenies. In parallel studies, *hc187* males reared at 25°C were found to have defects in sperm transfer (Figure 5D), a deleterious consequence of precocious male sperm activation that has been previously reported for both *swm-1* males (Stanfield and Villeneuve 2006) and suppressor alleles of *spe-4*, *spe-46*, and *spe-47* (Gosney *et al.* 2008; Liau *et al.* 2013; LaMunyon *et al.* 2015). (4) Analysis of hermaphrodite self-fertility revealed that the number of progeny produced by *spe-6 (hc187); him-8* hermaphrodites that were upshifted to 25°C before the L4 larval period of spermatogenesis [91.4 ± 6.1 (SEM); *n* = 16] was not statistically different from *him-8* controls [116.6 ± 5.9 (SE.); *n* = 9] or *hc187; him-8* hermaphrodites that had either been maintained at 16°C [119.2 ± 6.5 (SEM); *n* = 14] or upshifted to 25°C only after the L4 larval period of spermatogenesis [153.7 ± 5.8 (SEM); *n* = 14]. This analysis confirms that precocious sperm activation does not preclude hermaphrodite self-fertility. (5) Lastly, *spe-6(hc187)* was found to suppress the hermaphrodite self-sterility defect of *spe-29*(*it127*), a nontemperature-sensitive mutant of the *spe-8* group, at 25°C but not at 15°C (Figure 5D). Intriguingly, *hc187* partially suppressed the self-sterility of *spe-29(it127)* hermaphrodites at 20°C suggesting that at 20°C, SPE-6 levels are low enough to partially bypass the loss of *spe-29* in hermaphrodite sperm yet still high enough to prevent precocious activation of male sperm within the seminal vesicle.

### SPE-6(*hc163*) exhibits stage-specific defects in localization, but the mislocalized protein does not impair heterozygous sperm

**Figure 6:**
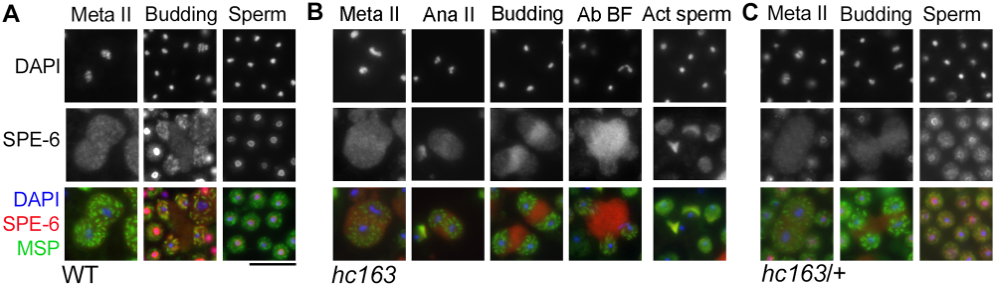
*spe-6*(*hc163*) produces a mislocalized protein product. (A–C) Meiotic spermatocytes and sperm from wild type, *spe-*6(*hc163*), and *hc163*/+ males labeled with DAPI (blue) and antibodies against SPE-6 (red) and MSP(green). Metaphase II (meta II), budding figure (budding), sperm (unactivated spermatid), anaphase II (ana II), Ab BF (abnormal budding figure), and a mix of activated and potentially unactivated sperm from mutant males (act sperm). Scale bar = 10 µm.

In contrast to *spe-6*(*hc187*), males with the canonical *spe-6*(*hc163*) allele produce stable and abundant SPE-6 protein; however, the *hc163* version of SPE-6 fails to block precocious sperm activation (Figure 4, B–E). To further explore how a valine to glutamine substitution within the conserved residue of the SPE-6 N-terminal ATPase domain impacts SPE-6, we examined the patterns of SPE-6 localization throughout spermatogenesis in *hc163* males (Figure 6). In gonad monolayer preparations from wild type and *spe-6*(*hc163*) males, the SPE-6 patterns in developing and meiotically dividing spermatocytes were largely indistinguishable (Figures 4B and 6, A and B). However, as spermatocytes transitioned from anaphase II to the postmeiotic process of RB formation (the budding division), they exhibited clear differences in the proportion of SPE-6 that partitioned to the RB. As previously noted (Figure 3) although a cytosolic fraction of SPE-6 partitioned to the RB during wild-type spermatogenesis, much of the particulate fraction partitioned to the sperm (Figure 6A). In contrast, most of the *hc163* form of SPE-6 protein partitioned to the RB, thus accounting for reduced levels in mutant sperm (Figure 6B). SPE-6(*hc163*) that does partition to the spermatids localizes to the cytosol of unactivated spermatids or to the pseudopods of spermatozoa. Notably, SPE-6(*hc163*) does not localize to the perinuclear halo (Figures 4B and 6B). Thus, the *hc163* lesion disrupts the protein’s proper partitioning during the budding division and its subsequent localization to the perinuclear halo, but the mutant protein retains functionality in the contexts of the sperm meiotic divisions and its ability to ultimately localize to pseudopods.

From these studies, it was unclear whether the *hc163* phenotype stems primarily from an overall reduction of SPE-6 levels within spermatids or the neomorphic consequence of SPE-6(*hc163*) being in the cytoplasm rather than localizing to the perinuclear halo (Figure 6C). To address the second possibility, we examined the sperm of *hc163* heterozygotes. Although *spe-6*(*hc163*) heterozygotes are phenotypically wild type for both regulated sperm activation in males and suppression of *spe-8* class alleles in hermaphrodites (Muhlrad and Ward 2002), we hypothesized that the wild-type protein might correct the localization of the mutant SPE-6 protein through a physical interaction between the two allelic forms. However, spermatids from these heterozygotes exhibit a combined pattern of both perinuclear and cytoplasmic SPE-6, indicating that the two allelic forms localize independently of the other. These findings are inconsistent with SPE-6(*hc163*) functioning neomorphically since cytosolic SPE-6(*hc163*) within the heterozygous spermatids is not sufficient to trigger precocious sperm activation. Instead, these results confirm that SPE-6(*hc163*) is phenotypically recessive for its function in blocking sperm activation.

### During the sequential process of sperm activation, SPE-6 relocalizes after spike formation and independently of MO fusion

**Figure 7:**
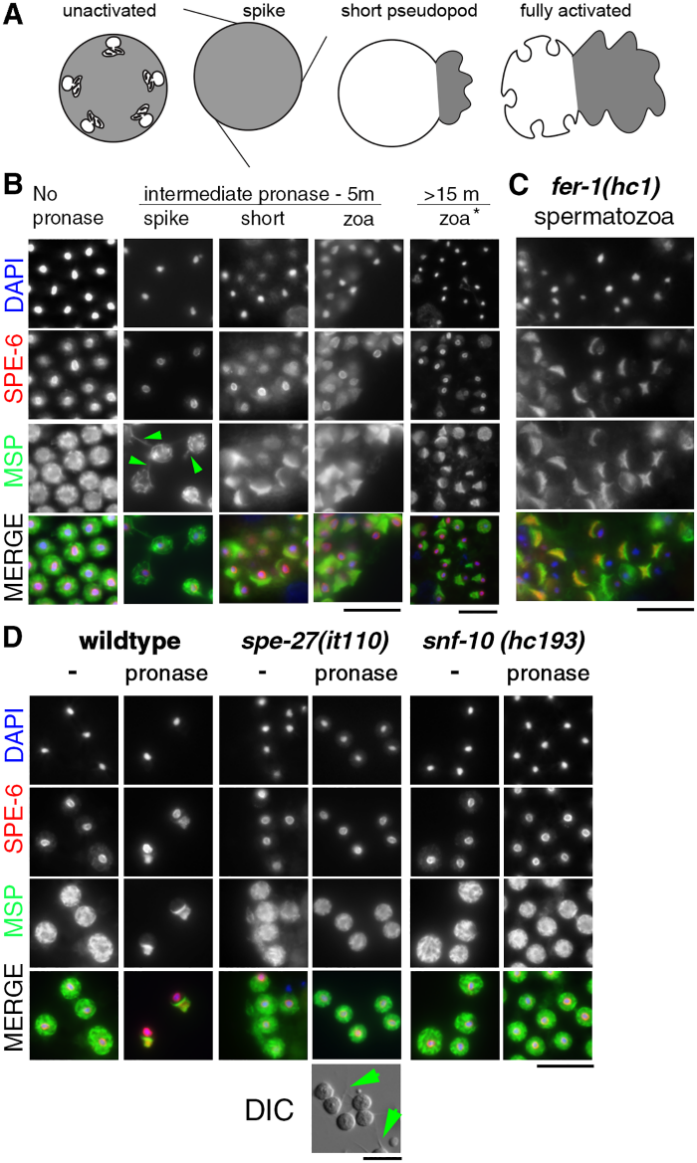
Release of SPE-6 from its perinuclear halo occurs after spike formation but independent of MO fusion. (A) Schematic of the substage of sperm activation. In unactivated sperm, MOs are docked with the plasma membrane. In fully activated spermatozoa, MOs have fused but retain a permanent fusion pore. Gray highlights MSP distribution. (B) Sperm from celibate males activated *in vitro* with pronase and labeled with DAPI (blue) and antibodies against SPE-6 (red) or MSP (green). Specific images show cells treated either for 5 min 20 sec. (intermediate) or for 15 min 34 sec. (>15 m). (C) Spermatozoa from MO-fusion defective, *fer-1*(*hc1*) mutants. (D) MSP (green) and SPE-6 (red) patterns in untreated or pronase treated sperm from the celibate males of wild type and activation pathway mutants. DIC image showing spikes (green arrowheads). Scale bars = 10 µm

During *C. elegans* sperm activation; immotile, spherical spermatids become motile spermatozoa with extended pseudopods. Studies of *in vitro* sperm activation, using a variety of drugs and proteases (Ward *et al.* 1983), have identified a spike-stage intermediate, during which multiple, filopodia-like, MSP-containing structures extend from all sides of the cell before coalescing into a single polarized pseudopod (Shakes and Ward 1989; Figure 7A). To better understand how SPE-6 functions in the context of sperm activation, we investigated whether SPE-6 is released from the perinuclear halo, before or after this spike stage. To do this, unactivated spermatids from celibate, wild-type males were treated with pronase, and then the samples were processed for anti-SPE-6 and anti-MSP immunofluorescence at various times during the 10- to 20-min activation process (Figure 7B). Since the exact timing of events often varied, we assessed SPE-6 patterns relative to the morphological characteristics of activation intermediates. In some preparations, the thin, transient spikes survived fixation and staining protocols and were detectable with MSP staining (Figure 7B, green arrowhead). In these spike stage cells, SPE-6 remained completely perinuclear just like unactivated spermatids. At intermediate time points, we also observed sperm with short, partially extended pseudopods (Figure 7B, “short”). In these “short” pseudopod sperm, MSP had fully compartmentalized to the pseudopod; however, very little SPE-6 had redistributed to the pseudopod. At later time points, even fully motile spermatozoa with long pseudopods (Figure 7B, “zoa”) retained high levels of perinuclear SPE-6 even as their pseudopod levels were increasing. These results suggest that spikes form before the release of SPE-6 from the perinuclear halo and that both pseudopod extension and sperm motility occur with only a partial redistribution of SPE-6 from the perinuclear halo. As observed in naturally activated spermatozoa (Figure 3), when pseudopods were present, the pseudopods always contained some amount of SPE-6. However, most spermatozoa also retained perinuclear SPE-6, indicating that the mere presence of SPE-6 in the perinuclear halo is insufficient to suppress pseudopod extension or sperm motility.

The other major feature of nematode sperm activation is the fusion of Golgi-derived MOs with the plasma membrane (Figure 6A). This nonreversible process is analogous to the acrosome reaction (Gleason *et al.* 2012) and results in both the extracellular release of soluble glycoproteins and the incorporation of key fertilization factors into the plasma membrane (reviewed in Krauchunas *et al.* 2016). To test whether MO fusion is necessary for SPE-6 relocalization, we analyzed SPE-6 patterns in *fer-1*(*hc1*) mutant sperm which activate to form short stubby pseudopods but fail to fuse their MOs and remain fertilization incompetent (Ward 1981). In spermatozoa from mated *fer-1* males, the stubby pseudopods labeled robustly with SPE-6 indicating that MO fusion is not required for SPE-6 to redistribute to the pseudopod (Figure 7C).

### During *in vitro* activation of male sperm, SPE-6 functions downstream of SNF-10 and SPE-8

Unclear from our analysis is whether SPE-6 relocalizes as part of the signaling process or as a downstream consequence of sperm activation. We therefore sought to assess SPE-6 localization in sperm that had initiated their activation signaling pathway but were unable to fully or robustly activate. Previous studies have shown that both SNF-10 and members of the SPE-8 signaling pathway are necessary but insufficient for sperm activation by pronase (Shakes and Ward 1989; Fenker *et al.* 2014; Tajima *et al.* 2019). When wild-type sperm are activated *in vitro*, activation typically occurs within 20 min after pronase treatment. However, key activation pathway mutants exhibit distinct responses. *spe-8* class sperm arrest in an intermediate spike state (Shakes and Ward 1989), whereas *snf-10* sperm exhibit no obvious morphological changes (Fenker *et al.* 2014). To determine whether Pronase treatment releases SPE-6 from an exclusively perinuclear localization pattern even in the absence of pseudopod formation, spermatids were isolated from wild type and mutant males and either directly processed for immunocytology or first treated *in vitro* with pronase (Figure 7D). In untreated wild-type spermatids, SPE-6 was perinuclear and MSP was cytosolic; but after pronase activation, MSP localized to the pseudopod and SPE-6 distributed between the pseudopod and the perinuclear halo. As expected, Pronase treatment of spermatids from the *spe-8* class mutant *spe-27*(*it110*) induced a spike stage arrest. In such sperm, SPE-6 remained strictly perinuclear (Figure 7D), similar to its pattern in both unactivated and wild-type spike stage intermediates (Figure 7B). In spermatids from *snf-10*(*hc193*) males, Pronase treatment likewise induced no change in the perinuclear localization of SPE-6 (Figure 7D). Thus for these mutants, SPE-6 localization patterns neither provide evidence of a novel activation intermediate nor serve as a marker that can distinguish between the Pronase responses of *spe-27 and snf-10* spermatids.

### Within the female reproductive tract, spermatozoa exhibit region-specific SPE-6 patterns

In both *in vivo* (Figure 3, D and E) and *in vitro* (Figure 7B) activated spermatozoa, the patterns of SPE-6 localization were surprisingly variable. Although some SPE-6 was always present in the pseudopods, the cells varied both in the proportion of SPE-6 remaining in the perinuclear halo and in the extent to which SPE-6 distributed throughout the pseudopod. To explain this variability, we considered two distinct models. Perhaps SPE-6 levels within the pseudopod increase over time, and newly activated spermatozoa always have a smaller proportion of SPE-6 in their pseudopods. Alternatively, these distinct patterns might reflect important physiological differences between individual spermatozoa. Although the self-fertility of restrictively grown *spe-6(hc187)* hermaphrodites indicates that SPE-6 is not essential for spermatozoa function, SPE-6 could still function, perhaps redundantly with other kinases, to optimize the physiology of spermatozoa, postinsemination.

**Figure 8:**
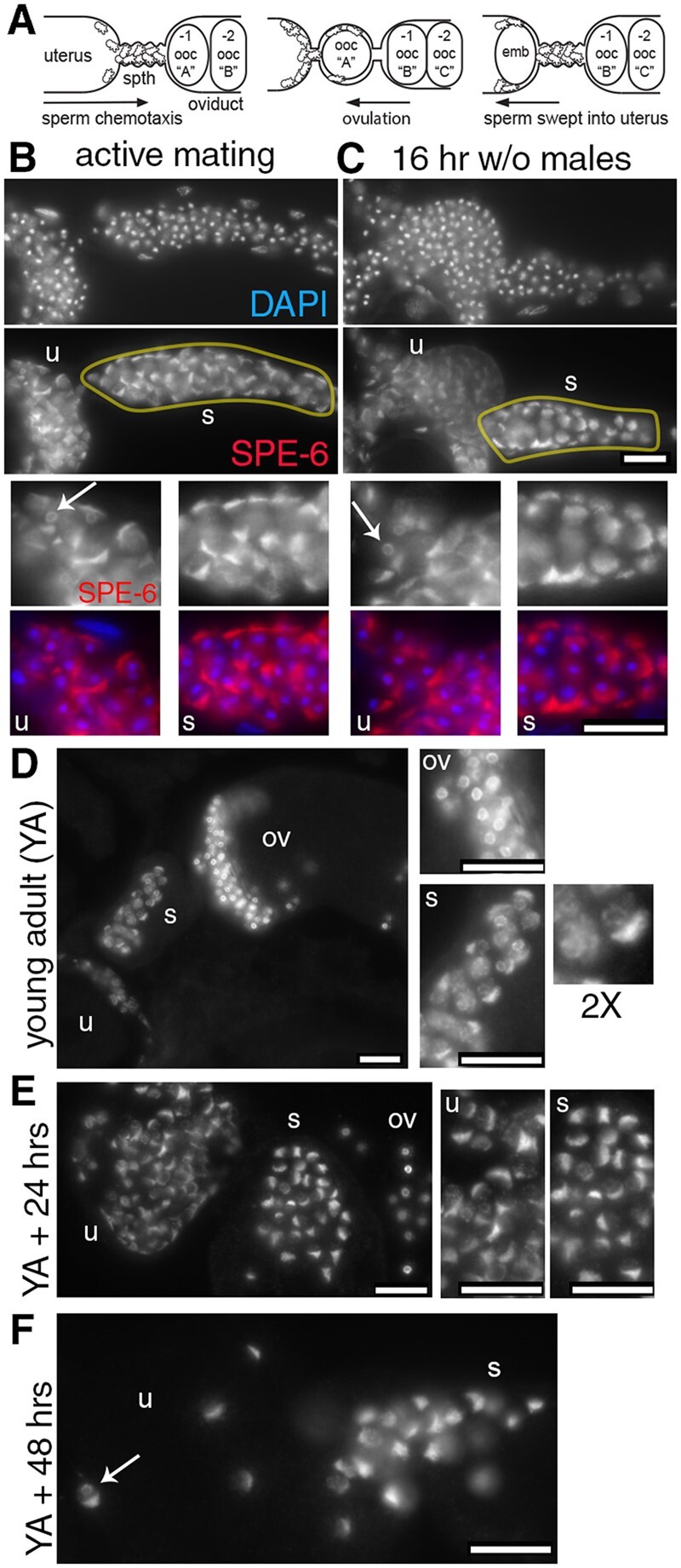
Within the female reproductive tract, wild-type spermatozoa exhibit regional-specific SPE-6 localization patterns. (A) Schematic of male sperm movements within the female reproductive tract. Newly inseminated sperm actively chemotax from the uterus to the spermatheca, which serves the dual function of storing sperm and serving as the site of fertilization. The movement of the sperm into the spermatheca is counterbalanced by the physical displacement of the sperm as oocytes enter one-by-one into the spermatheca and the newly fertilized embryos pass into the uterus. In a cyclical process, sperm that have been displaced into the uterus must migrate back into the spermatheca. (B, C) Male spermatozoa within the isolated, yellow encircled spermatheca (s) and uterus (u) from *fog-2* females that were either actively mating (B) or had been isolated from males for 16 h (C). Full size SPE-6 only or merge images in the lower panels show regions of the uterus or spermatheca. (D–F) Hermaphrodite sperm within the reproductive tract of a very young, wild-type adult (D) and older 24 h (E) and 48 h (F) postmolt hermaphrodites. In full-sized images and 2X enlargement, SPE-6 is perinuclear in the unactivated spermatids within the oviduct (ov) but within the spermatozoa in the uterus and spermatheca, SPE-6 is either confined to the pseudopod or in partially perinuclear and cytoplasmic. Specimens were labeled with DAPI (blue) and anti-SPE-6 (red). White arrows indicate spermatozoa with perinuclear SPE-6. Scale bars = 10 µm.

Sperm migration patterns within the female reproductive tract of *C. elegans* are complicated, even when focusing specifically on male sperm (Figure 8A; Ward and Carrel 1979). During mating, male sperm are initially deposited through the vulva into the uterus. Sperm activation occurs within the uterus (Smith and Stanfield 2011), and the newly motile spermatozoa crawl toward the spermatheca. However, spermatozoa that had previously reached the spermatheca are often forcibly displaced back into the uterus either as oocytes from the oviduct enter the spermatheca and distend the spermathecal wall or as newly fertilized embryos pass from the spermatheca into the uterus. Thus, the ability of every nematode sperm to participate in a fertilization event requires individual sperm to undergo multiple rounds of migration from the uterus back into the spermatheca.

To better understand these variable SPE-6 patterns and how SPE-6 might function within spermatozoa, we examined the SPE-6 patterns of male spermatozoa within the hermaphrodite reproductive tract. For the first set of experiments, wild-type males were allowed to mate with *fog-2 “*females” for 24 h. These mated females were then either immediately processed for immunofluorescence or maintained on plates without males for 2–16 h before processing. Male spermatozoa within these mated females exhibited a range of SPE-6 patterns (Figure 8, B and C). Surprisingly, these patterns were regional biased. Sperm within the uterus exhibited proportionally higher levels of perinuclear SPE-6, while those within the spermatheca commonly had all or most of their SPE-6 confined to the pseudopod. Notably, we observed similar patterns, regardless of whether the females had been actively mating (Figure 8B) or had been separated from males for 16 h (Figure 8C).

In parallel studies, we observed similar, regional differences in hermaphrodite sperm. Very young, unmated adult hermaphrodites had both unactivated spermatids in the oviduct and spermatozoa in the spermatheca (Figure 8D). Within unactivated spermatids, SPE-6 was exclusively perinuclear. Within spermatozoa, a portion of the SPE-6 pool localized to the pseudopod, but the remainder was in the cell body, mostly in distinct cytoplasmic puncta and sometimes also perinuclear (Figure 8D). In older animals, sperm in the spermatheca had less perinuclear SPE-6, but SPE-6 remained enriched in the pseudopod and cytoplasmic puncta (Figure 8, E and F).

### *In vivo* SPE-6 patterns within sperm activation mutants

**Figure 9:**
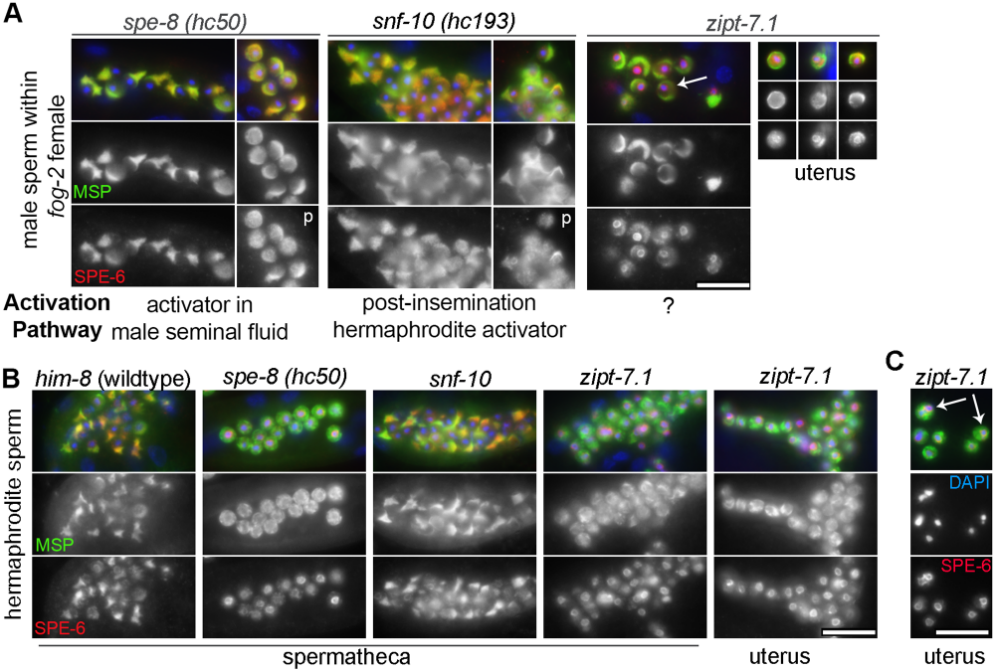
SPE-6 patterns in mutant sperm exposed *in vivo* to male and hermaphrodite sperm activators. (A) Male spermatozoa from the indicated mutant males within the reproductive tract of *fog-2* females. Except for the individual *zipt-7.1* sperm that were observed within the uterus (far right), all of the imaged sperm were present in the spermatheca. Some male spermatozoa include SPE-6 in the form of cytoplasmic puncta (p). Some *zipt-7.1* sperm had bipolar pseudopods (arrow). (B) Hermaphrodite sperm within the spermatheca or uterus of the indicated mutants. (C) Examples of sperm size variation and chromatin fragmentation (arrow) amongst *zipt-7.1* sperm. Specimens were labeled with DAPI (blue), anti-SPE-6 (red), and anti-MSP (green). Scale bars = 10 µm.

To extend our *in vitro* studies of sperm activation mutants (Figure 7), we next examined their *in vivo* SPE-6 patterns. To do this, we first compared male spermatozoa within *fog-2* “females” that had been crossed with either *spe-8* (disrupted hermaphrodite pathway) or *snf-10* (disrupted male pathway) males (Figure 9A). Consistent with previous studies (Shakes and Ward 1989; Fenker *et al.* 2014), both crosses yielded progeny as the male sperm could be activated by the alternative, nondisrupted pathway. In both cases, the SPE-6 patterns were similar to those of wild-type male spermatozoa except that SPE-6 cytoplasmic puncta were more common.

Next, we examined *zipt-7.1* male sperm. *zipt-7.1* is a zinc transporter that localizes to MOs and is required for full fertility in both hermaphrodites and males (Zhao *et al.* 2018; Figure 1B). ZIPT-7.1 is proposed to function downstream of SPE-6 since *spe-6(hc163)* fails to suppress the self-infertility of *zipt-7.1* hermaphrodites (Zhao *et al.* 2018). To determine whether the low fertility of *zipt-7.1* males might be associated with altered SPE-6 patterns, we examined *zipt-7.1* male sperm within *fog-2* “females.” Only small numbers of *zipt-7.1* male spermatozoa could be found in either the spermatheca or uterus, suggesting a defect in either sperm transfer or retention. *zipt-7.1* sperm that were present in the spermatheca had abnormally broad, occasionally bipolar (arrow) pseudopods, and SPE-6 localized to both their pseudopods and perinuclear regions (Figure 9A). *zipt-7.1* sperm within the uterus had similar SPE-6 patterns but even broader and more reduced pseudopods which presumably compromise their directed cell motility.

Lastly, we examined *in vivo* activated hermaphrodite sperm in unmated young adult mutant hermaphrodites (Figure 9B). As expected, *spe-8* sperm failed to activate and retained spermatid patterns of MSP and SPE-6 whereas *snf-10* sperm successfully activated via the hermaphrodite activation pathway and exhibited spermatozoa patterns of MSP and SPE-6 that were indistinguishable from *him-8* controls. In *zipt-7.1* hermaphrodites, we expected based on previous studies (Zhao *et al.* 2018) that the sperm would fail to extend pseudopods, but if ZIPT-7.1 functions downstream of SPE-6, then upstream signaling from SPE-8 might trigger a partial redistribution of SPE-6 from the perinuclear halo to cytoplasmic puncta. However, no such patterns were observed, and SPE-6 remained exclusively perinuclear. Together these results provide new insights into the function of ZIPT-7.1. They are consistent with models in which ZIPT-7.1 functions downstream of the SPE-8 but less directly in the SNF-10 pathway in the context of the sperm activation (Zhao *et al.* 2018). Furthermore, the cytology of the *in vivo* activated male sperm may suggest an additional role for ZIPT-7.1 in regulating the cell polarity of already activated spermatozoa.

## Discussion

Previous studies identified a requirement for SPE-6 in packaging sperm motility proteins within sperm-specific FB-MOs, supporting proper progression through the meiotic divisions, and blocking premature sperm activation. To investigate how SPE-6 functions in these diverse processes, we studied the localization of SPE-6 protein at distinct stages of sperm development. Consistent with SPE-6 functioning in multiple processes; SPE-6 localization is highly stage-specific, and SPE-6 relocalizes during key transitions in sperm development and function. Prior to and during the meiotic divisions, SPE-6 protein distributes in a speckled cytosolic pattern. In haploid spermatids, SPE-6 relocalizes to the “perinuclear” region surrounding the compacted sperm chromatin mass. Following sperm activation, SPE-6 distributes between the perinuclear compartment and the pseudopod in a manner that loosely correlates with the physical localization of the sperm within the hermaphrodite reproductive tract. To our knowledge, no other protein in *C. elegans* sperm has been described as having a potentially dynamic relocalization pattern within spermatozoa. Indeed, two other proteins implicated in both FB-MO formation and the negative regulation of sperm activation, SPE-47 and SPE-50, are completely absent from haploid spermatids (Clark *et al.* 2020), while a third protein, SPE-4, maintains an MO-associated pattern throughout sperm development and activation (Arduengo *et al.* 1998, Gosney *et al.* 2008). While the direct molecular targets, interactors, and upstream regulators of SPE-6 remain to be identified, these intriguing localization patterns coupled with the highly penetrant *spe-6* mutant phenotypes suggest unappreciated complexities in the regulation of both the initial irreversible sperm activation event and in subsequent events that govern sperm motility.

Within developing spermatocytes, SPE-6 is expressed during early pachytene, later than transcriptional regulators that implement the sperm program (Kulkarni *et al.* 2012; Ragle *et al.* 2020) but prior to many sperm function proteins. Within individual spermatocytes, SPE-6 is present in both a diffuse cytosolic pool as well as a structured particulate pool. The nature of the SPE-6 particulate pool remains unclear. A similar pattern was previously reported for COMP-1 (Hansen *et al.* 2015), and it is possible that both localize to a previously undescribed membrane-less compartment. However, given that SPE-6 is critical for both FB assembly and meiotic progression beyond prometaphase I (Varkey *et al.* 1993), these complex and potentially dynamic patterns are consistent with SPE-6 functioning in multiple and diverse cellular pathways. Our discovery that SPE-6 does not localize specifically to FB-MOs raises the possibility that SPE-6 either functions indirectly to promote FB-MO formation or that SPE-6 dynamically localizes to the FB-MO complex without actually accumulating there.

Within unactivated spermatids, SPE-6 appears to localize exclusively to the perinuclear halo. This perinuclear compartment was initially described in ultrastructural studies as electron dense, RNA rich, and enveloping the quiescent sperm centrioles (Ward *et al.* 1981). Another protein that localizes to the perinuclear halo is the paternal effect protein SPE-11 which is required for postfertilization egg activation events (Hill *et al.*1989; Browning and Strome 1996). Like SPE-6, SPE-11 distributes to cytoplasmic granules in mutant sperm that lack chromosomes (Sadler and Shakes 2000). Proper assembly of the perinuclear halo requires the E3 ubiquitin ligase mindbomb (Herrera and Starr 2018; Ratliff *et al.* 2018) and the 26G-RNA-related Argonaut proteins ALG-3/4 (Conine *et al.* 2013). Thus our discovery that SPE-6 localizes to the perinuclear halo was surprising since this location places SPE-6 amongst components needed for postfertilization functions and physically apart from known components of the sperm activation pathways. On the other hand, as a factor whose repression is proposed to occur downstream of core activation components, SPE-6 function could be plausibly modulated by either the nonreceptor tyrosine kinase SPE-8 as it is released from the plasma membrane (Muhlrad and Ward 2002) or secondary messengers related to changes in cellular pH (Ward *et al.* 1983) or zinc ions (Zhao *et al.* 2018). While some members of the CK1 superfamily are regulated by post-translational modifications, most are thought to be regulated by their localization through binding partners which either sequester them from or bring them in close proximity to their substrates (Fulcher and Sapkota 2020).

Previous studies suggested a model in which SPE-6 functions as a brake within spermatids to inhibit sperm activation (Muhlrad and Ward 2002; Liau *et al.* 2013). Our results support this model by showing that SPE-6 is indeed present in sperm and that a temperature-sensitive reduction of SPE-6 (*hc187*) protein levels results in precocious sperm activation. Furthermore, the localization defects of *spe-6(hc163)* help explain why *hc163* is the most robust suppressor allele of *spe-27(it132).* By discarding SPE-6 protein only after the meiotic divisions, *spe-6(hc163)* sperm have sufficient SPE-6 for the protein’s early functions within spermatocytes, yet are poised to precociously activate in spermatids which are both depleted of SPE-6 and fail to properly localize the small amounts of SPE-6(hc163) that do make it into the spermatids. At the same time, the finding that the very low level of SPE-6 in restrictively grown *spe-6(hc187)* spermatocytes can successfully assemble FBs and complete meiosis, suggests that only minimal catalytic amounts of SPE-6 are required for these early developmental functions.

Our studies do not support simple models in which perinuclear SPE-6 represents a “brake on” state or fully released SPE-6 triggers activation. In *spe-6*(*hc163*) heterozygotes, the nonperinuclear fraction is insufficient to trigger precocious activation (Figure 6). Conversely, the significant amounts of SPE-6 that remain perinuclear in activating or recently activated wild-type sperm indicate that the mere presence of perinuclear SPE-6 is insufficient to repress activation (Figures 7 and 8D). At least small amounts of SPE-6 leave the perinuclear store to colocalize with MSP during the earliest stages of pseudopod formation but not during the preceding spike stage of either wild-type activation intermediates or pronase activated SPE-8 class sperm. Notably, localization of SPE-6 to the developing pseudopod can occur independently of MO fusion, suggesting that the relocalization of SPE-6 correlates directly with pseudopod formation rather than secondarily with MO fusion. Thus, while having the cell’s entire SPE-6 pool confined to the perinuclear halo may correlate with a functional SPE-6 brake, repressing the braking function of SPE-6 is likely to involve changes in SPE-6 enzyme activity or its interactors that operate at a finer scale than relocalization of the entire SPE-6 pool.

Within spermatozoa, localization of SPE-6 to the leading edge and center of the pseudopod suggests exciting connections to studies of Ascaris sperm (reviewed in Roberts and Stewart 2012) and *C. elegans* studies of the PP1 phosphatase GSP3/4 (Wu *et al.* 2012). In these collective studies, the assembly/disassembly dynamics of MSP within crawling spermatozoa is regulated by kinase activity at the leading edge and phosphatase activity at the pseudopod/cell body interface. Here, we show that SPE-6 is often confined to a subregion of the pseudopod of actively mobile spermatozoa, namely those that were newly activated or present in the uterus. This pattern may suggest that SPE-6 contributes directly to MSP dynamics within crawling spermatozoa. Yet if SPE-6 plays such a role, it must function redundantly as SPE-6 depleted *hc187* sperm undergo normal MSP dynamics.

An exciting but unexpected finding is that SPE-6 is not strictly localized to the pseudopod of spermatozoa but instead distributes between the pseudopod, perinuclear halo, and cytoplasmic puncta in a manner that loosely correlates with its position within the female reproductive tract. Spermatozoa within the uterus or within younger animals typically had more perinuclear SPE-6 while spermatozoa in within the spermatheca of older, less frequently ovulating females typically had SPE-6 confined largely or exclusively to the pseudopod. These findings are the first to suggest molecular differences between nematode spermatozoa depending on their location within the female reproductive tract, and they suggest that SPE-6 patterns may provide a useful marker for these changes.

It is important to remember that although SPE-6 is in the right place at the right time to contribute to a multitude of functions within crawling spermatozoa, *spe-6(hc163) and spe-6(hc187)* hermaphrodites are fertile while defects in male fertility reflect a problem in the effective transfer of precociously activated sperm. Loss-of-function defects in SPE-6 do not dramatically compromise either sperm motility or chemotaxis within the female reproductive tract. On the other hand, given the large number of SPE-6 like genes in the *C. elegans* genome, it would not be surprising if some potential SPE-6 functions, particularly within crawling spermatozoa, are carried out redundantly such that loss of function phenotypes will require multigene knockdowns. As *C. elegans* continues to serve as an important model for understanding the molecular mechanisms of sperm guidance (Han *et al.* 2010; Hu *et al.* 2019) and competition (Hansen *et al.* 2015) within the female reproductive tract, SPE-6 promises to be an exciting new marker of “under the hood” spermatozoa physiology.

## Data availability

Strains and antibodies are available upon request. The authors affirm that all data necessary for confirming the conclusions of the article are present within the article, figures, and tables.

Supplementary material is available at *G3* online.

## Supplementary Material

jkab288_Supplementary_Data
